# Supplementary material for: A Model H5N2 Vaccine Strain for Dual Protection Against H5N1 and H9N2 Avian Influenza Viruses
Source: Vaccines (Basel). 2024 Dec 30;13(1):22. doi: 10.3390/vaccines13010022 (PMC11768808; doi:10.3390/vaccines13010022)
Supplement: Supplementary file 1 [file vaccines-13-00022-s001.zip › Supplementary file _A model H5N2 vaccine strain for dual protection against H5N1 and H9N2 avian influenza viruses.pdf]

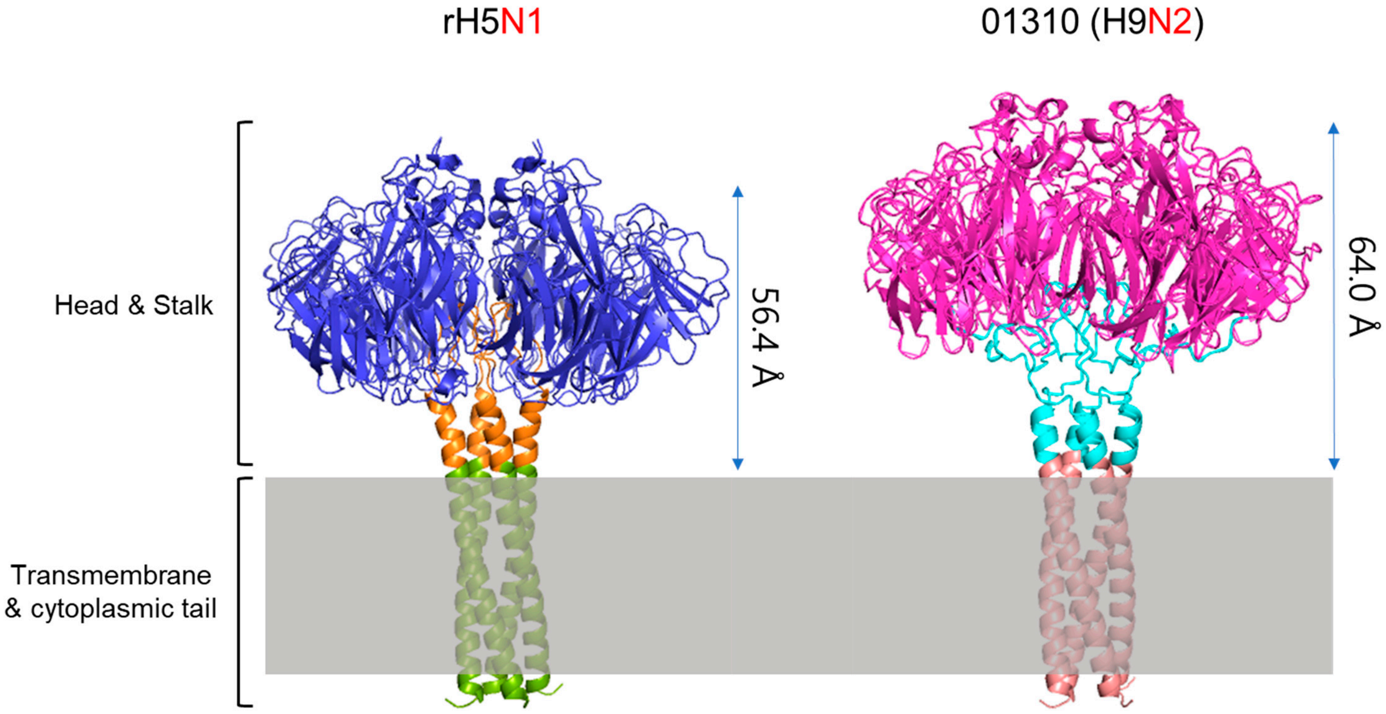

Figure S1. Comparison of the ectodomain lengths of N1 in rH5N1 and N2 in 01310 (H9N2) Using the AlphaFold 3 model, we predicted the structures of the N1 protein from the K10-483 virus (rH5N1) and the N2 protein from the 01310 virus. The predicted structures were visualized with PyMOL v4.6.0. The average distance among the five conserved active-site amino acids (R118, R152, E276, R292, R371) present in all the influenza viruses and the amino acid positioned lowest in the stalk was measured to compare the heights of N1 in the K10-483 virus and N2 in the 01310 virus.

Table S1. Selection of the M2e (Av) sequence. The M2e sequences of H5N1, H5N6, H5N8, and H9N2 avian influenza viruses isolated between 2018 and 2021 were aligned, and the M2e (Av) sequence was selected as the sequence that matched the consensus sequence without any potential N-glycosylation site.

	**M2e sequence (1-24)**
**Consensus sequence**	**MSLLTEVETPTRNGWECNCSDSSD**
M2e (Av) sequence	.................K...... [b]
H5N1 (n=627[a])	...........K............
	...........KK...........
H5N6 (n=364)	.........L.KT.......G...
	.................K......
	...........K............
	............T.......G...
	.................R......
H5N8 (n=1453)	...........K............
	....
	....N......K............
	.................K......
H9N2 (n=993)	............T.......G...
	.........L.KT.......G...
	.........L..T.......G...
	.........L.K........G...
	.........L..............
A/Puerto Rico/8/1934 (PR8, H1N1)	..........I..E.G.R.NG.[c]..

[a] Number of sequences used for comparison

[b] Amino acid sequences that are identical to the consensus sequence are denoted with a dot.

[c] The underlined text in red indicates a potential N-glycosylation site, represented by the motif Asn-X-Ser/Thr (where X can be any amino acid except proline).

Table S2. **Matching CD8+ T-cell epitopes of the vaccine and challenge strains.**

Strain	HA (SNU50-5 numbering)															
	HA1 subunit										HA2 subunit					
	22-32	113-121	125-133	158-166	179-189	204-214	212-221	256-264	307-315	320-327	435-443	452-460	460-468	525-535	531-539	550-558
VS*	GYHANNSTEQV	DYEELKHLL	NHFEKIQII	SFFRNVVWL	GYNNTNQEDLL	TRLYQNPTTYI	TYISIGTSTL	HFESNGNFI	FHNIHPLTI	KYVKSNKLV	TYNAELLVL	FHDSNVKNL	LYDKVRLQL	IYQILSIYSTV	IYSTVASSL	
SNU50-5	GYHANNSTEQV	DYEELKHLL	NHFEKIQII	SFFRNVVWL	SYNNTNQEDLL	TKLYQNPTTYV	TYVSVGTSTL	NFESNGNFI	FHNIHPLTI	KYVKSDRLV	TYNAELLVL	FHDSNVKNL	LYDKVRLQL	TYQILSIYSTV	IYSTVASSL	FWMCSNGSL
PR8	GYHANNSTDTV	DYEELREQL		SFYRNLLWL	SYVNKKGKEVL	--LYQNENAYV		IFEANGNLI	YQNIHPVTI	KYVRSAKL	TYNAELLVL	FHDSNVKNL	LYEKVKSQL	IYQILAIYSTV	IYSTVASSL	FWMCSNGSL

Strain	NA (SNU50-5 numbering)						
	99-108	125-133	131-140	169-177	207-216	352-359	405-412
SNU50-5	IYSKDNGIRI	SHLECRTFF	-FFLTQGALL	PYNSRFESV	KYNGIITDTI	--KYGNGVWI	SFVQHPEL
PR8	IYSKDNSIRI	SHLECRTFF	-FFLTQGALL	PYNSRFESV	KYNGIITETI	SYRYGNGVWI	SFVQHPEL
VS			QFALGQGTTL		IYDGVLVDSI		

Strain	NP						M1				NEP		
	39-47	55-63	77-85	147-155	218-226	345-353	99-107	131-138	221-229	239-248	8-16	72-80	98-106
VS & PR8	FYIQMCTEL	RLIQNSLTI	KYLEEHPSA	TYQRTRALV	AYERMCNIL	SFIKGTKVL	LYRKLKREI	IYNRMGAV	THPSSSAGL	AYQKRMGVQM	SFQDILLRM	KFEEIRWLI	TFMQALHLL
SNU50-5	FYIQMCTEL	RLIQNSITI	KYLEEHPSA	TYQRTRALV	AYERMCNIL	SFIRGTRVV	LYRKLKREI	IYNRMGTV	THPSSSAGL	AYQKRMGVQM	SFQDILQRM	KFEEIRWLI	TFLQALQLL

* VS: vaccine strain (rH5N2 and rH5N2-aM2e)

Sequences that differ from those of the vaccine strains (rH5N2 and rH5N2-aM2e) are highlighted in red and bold text.
